# tidk: a toolkit to rapidly identify telomeric repeats from genomic datasets

**DOI:** 10.1093/bioinformatics/btaf049

**Published:** 2025-01-31

**Authors:** Max R Brown, Pablo Manuel Gonzalez de La Rosa, Mark Blaxter

**Affiliations:** School of Life Sciences, Anglia Ruskin University, Cambridge, CB1 1PT, United Kingdom; Tree of Life, Wellcome Sanger Institute, Hinxton, CB10 1RQ, United Kingdom; Tree of Life, Wellcome Sanger Institute, Hinxton, CB10 1RQ, United Kingdom

## Abstract

**Summary:**

“tidk” (short for telomere identification toolkit) uses a simple, fast algorithm to scan long DNA reads for the presence of short tandemly repeated DNA in runs, and to aggregate them based on canonical DNA string representation. These are telomeric repeat candidates. Our algorithm is shown to be accurate in genomes for which the telomeric repeat unit is known and is tested across a wide variety of newly assembled genomes to uncover new telomeric repeat units. Tools are provided to identify telomeric repeats *de novo*, scan genomes for known telomeric repeats, and to visualize telomeric repeats on the assembly. “tidk” is implemented in Rust and is available as a command line tool which can be compiled using the Rust toolchain or downloaded as a binary from bioconda.

**Availability and implementation:**

The “tidk” Rust crate is freely available under the MIT license (https://crates.io/crates/tidk), and the source code is available at https://github.com/tolkit/telomeric-identifier.

## 1 Introduction

Telomeric repeats are found at the ends of eukaryotic chromosomes, being generally composed of short, guanine-rich, tandemly repeated sequences (Bolzán and Bianchi 2006). They are functionally important primarily to ensure chromosomal stability during replication and are maintained by telomerase enzymes, or by recombination (Kurenova and Mason 1997, Abad *et al.* 2004). They generally have been described in the literature in the form T_n_A_n_G_n_, where G_n_ is frequently the dominant component of the repeat (Peska and Garcia 2020). When assembling genomes, it is important to characterize genome completeness. One measure of completeness is the presence or absence of telomeric repeats at the ends of the assembled chromosomal pseudomolecules.

Despite their importance, few tools exist to identify telomeric repeats bioinformatically, and most only search for known or user-specified single telomeric repeats. For example, “TelomereHunter” and “TelSeq” look only for the repeat “TTAGGG” (Lee *et al.* 2017, Feuerbach *et al.* 2019). Others use online platforms limited to a restricted set of kmers for analysis, e.g. SERF (Somanathan and Baysdorfer 2018), and hence lack flexibility.

To overcome these limitations, we present telomere identification toolkit (tidk), a fast command line tool for identifying, searching for, and visualizing telomeric repeats from fasta files. tidk predicts telomeric repeats *de novo* using a kmer-based approach. By prioritizing continuous runs of identical repeats and aggregating on canonical putative telomeric repeats using string rotation algorithms, tidk can quickly predict candidate telomeric repeats. This facilitates large scale and phylogenetically diverse assessments of telomeric repeat sequences, including clades lacking annotated telomeres as well as complex metagenomic assemblies where more than one telomeric repeat sequence might be found.

## 2 tidk implementation and usage

tidk is implemented in the systems programming language Rust, for speed and memory safety and the Rust-bio crate is used for fasta parsing and optimized pattern matching (Köster 2016). The input fasta files to tidk can be of any length. The executable “tidk” has five subcommands.

The first, “tidk explore” is used to discover putative telomeric repeats. The algorithm iterates over the whole genome in chunks of size k. Where tandemly repeated kmers occur of any multiple (i.e. ≥ 2), they are collapsed into consecutive runs. These runs are represented in memory as a vector of strings and position range indices. A threshold parameter is used to filter out short tandem repeat runs. The resulting putative telomeric repeats are stored in a hashmap, where each repeat serves as a key, and its frequency is the value. To ensure that equivalent repeats are not counted separately, the hashmap is further simplified by finding the lexicographically smallest representation of each repeat, considering all possible rotations and reverse complements. This process merges entries that are identical in different orientations, reducing redundancy (see Supplementary File S1 for pseudocode). Time complexity for the “tidk explore” is O(n) and space complexity is O(n), where n is the total length of input sequence in the fasta file. If there are many putative telomeric repeats, this may dominate time complexity due to the large number of pairwise comparisons in the last hashmap simplification step. This O(m^2^) computation however is usually a small part of the whole algorithm (where m is the number of unique sequences in the final hashmap), especially if the threshold parameter for consecutive repeat length is set to a sensible number (e.g. 50+).

The length of kmer to search for in “tidk explore” is specified as a mandatory variable input argument either as a single number, or a range. If a kmer length range is selected, then “tidk explore” will iteratively search the genome using each kmer length and attempt to parallelize record processing within the fasta file. Almost all canonical telomeric repeat units seen so far are <15 bp. The exceptions may be fungi and some plants. Hence, we recommend a kmer length between 4 and 15 for telomeric repeat discovery (Supplementary File S2). If no results are returned the threshold parameter can be lowered so that shorter tandem repeats are considered. A tab separated file (TSV) of potential telomeric repeats and their relative counts are printed to the standard output. Longer input sequences give better estimates and program performance. Using a range of kmer lengths from 5 to 30, performance is around 20 s per gigabase of input DNA on an Apple M1 Macbook Pro with 8 cores and 16 GB memory (see Supplementary File S2 for visual breakdown of performance over different genome sizes and kmer lengths).

“tidk search” and “tidk find” are subcommands that search for a specific repeat in the input fasta file. Both algorithms have a best-case time complexity of O(n) where n is the input sequence length, whilst space complexity is O(n) for both algorithms. Performance of these two subcommands is around 10 s per gigabase. In “tidk search,” the user specifies a string to search, and in “tidk find” a user chooses a clade to which their sequenced organism belongs to. Both subcommands use Rust-bio implemented pattern matching algorithms. If the input motif length is <65 characters, KMP [Knuth, Morris, and Pratt; time complexity O(n)] is used, else BOM [backward oracle matching; worst case O(n * m)] is executed (Köster 2016).

Additionally, a small database is provided based upon 500 chromosomally complete genome assemblies from the Darwin Tree of Life (DToL) project (Darwin Tree of Life Project Consortium 2022). This database should be downloaded using the “tidk build” subcommand. The output of “tidk search” and “tidk find” is a TSV text file which contains counts of matches in windows across the input fasta file. The output TSV can be used as input to the last subcommand, “tidk plot.” “tidk plot” generates an SVG file with line plots representing the telomeric repeat counts in windows across the sequence.

## 3 Limitations and error simulations

There are some known limitations in our toolkit. If the telomeric repeats contain high frequencies of higher order repeats (HORs), the “tidk explore” aggregation algorithm is less efficient. For example, if we had a consecutive sequence composed of a 2-mer of repeat one (TTAGG) and a 2-mer of repeat two (TTACG), TTAGGTTAGGTTACGTTACG, there would be fewer runs of any single consecutive kmer repeat, and greater uncertainty in what the repeat unit would be.

Error rates in telomeric sequences as well as their length impact the accuracy of the telomere identification. By simulating genomes, each with a thousand sequence replicates of varying lengths (600, 12 000, or 30 000 nucleotides) and composed of TTAGGG telomeric repeats (with the lexicographically minimal canonical telomeric repeat AACCCT) at different error rates (0%, 0.1%, 1%, 1.5%, 2%, 5%, and 10%), we aimed to assess the performance of “tidk explore” in identifying telomeric repeats. Our analysis revealed that the most abundant canonical repeat unit matched the simulated canonical repeat in 6 out of 21 conditions (though two more were dimers of the true repeat; see Supplementary Table S3). The true repeat (AACCCT) was not found at all in the output in 5 out of 21 conditions, however the error rate in these sequences was 2% or higher. The most abundant canonical repeat unit identified by “tidk explore” often missed just one nucleotide of the telomeric repeat, such as identifying ACCCT instead of AACCCT. This indicates that substrings of the motif are more likely to be identified as the most abundant repeat unit rather than the true telomeric repeat when the sequence has a high error rate. Consequently, manual validation is indispensable when selecting the telomeric repeat among the candidates shortlisted by “tidk explore.” Our results suggest that higher error rates and shorter sequence lengths negatively impact the performance of “tidk explore” in detecting canonical telomeric repeats de-novo.

In summary, while “tidk explore” shows promise in identifying telomeric repeats de-novo, its performance is influenced by the error rate and sequence length. The tool tends to identify substrings of the canonical repeat when errors are present, highlighting the need for careful manual validation in sequences with significant fraction of errors to ensure accurate identification of canonical telomeric repeats units. If the genome contains very long telomeric repeat units (as in some fungi) or the telomeres are composed of alternative structures as in Drosophila and most flies, or HORs, tidk will not be able to find the canonical repeat unit in most cases.

## 4 Predicting telomeric repeats across 500 reference genomes

The DToL project (Darwin Tree of Life Project Consortium 2022) aims to sequence to chromosomal level the genomes of all eukaryotic species in the UK and Ireland (see https://darwintreeoflife.org). To illustrate the use of tidk we used it to generate a small database containing predicted canonical telomeric repeats for 500 taxa sequenced by DToL. The phylogenetic range of these 500 species is large, encompassing plants (Streptophyta), animals (Arthropoda, Annelida, Chordata, and others), protists (Apicomplexa), and fungi (Mucoromycota). From these 500 genomes, we recovered putative telomeric repeats from 421 species (see Data and Software Availability). For chordates, we recovered the lexicographically minimal canonical telomeric repeat (AACCCT; Podlevsky *et al.* 2008) for all 21 species analyzed. This is equivalent to the known repeat TTAGGG, reverse complemented, and string rotated. A variant of this repeat, AACCT, was found in 308 species within the Arthropoda, and constituted the most abundant canonical telomeric repeat for genomes in our dataset. Almost all flowering plants analyzed (19/20) had the repeat AAACCCT, another well-known repeat variant found in plants. Omitting these simple variations left 45 species for which the telomeric repeats were more complex and variable. The largest taxon group carrying these complex repeats was the Hymenoptera, which we analyzed further.

**Figure 1.**
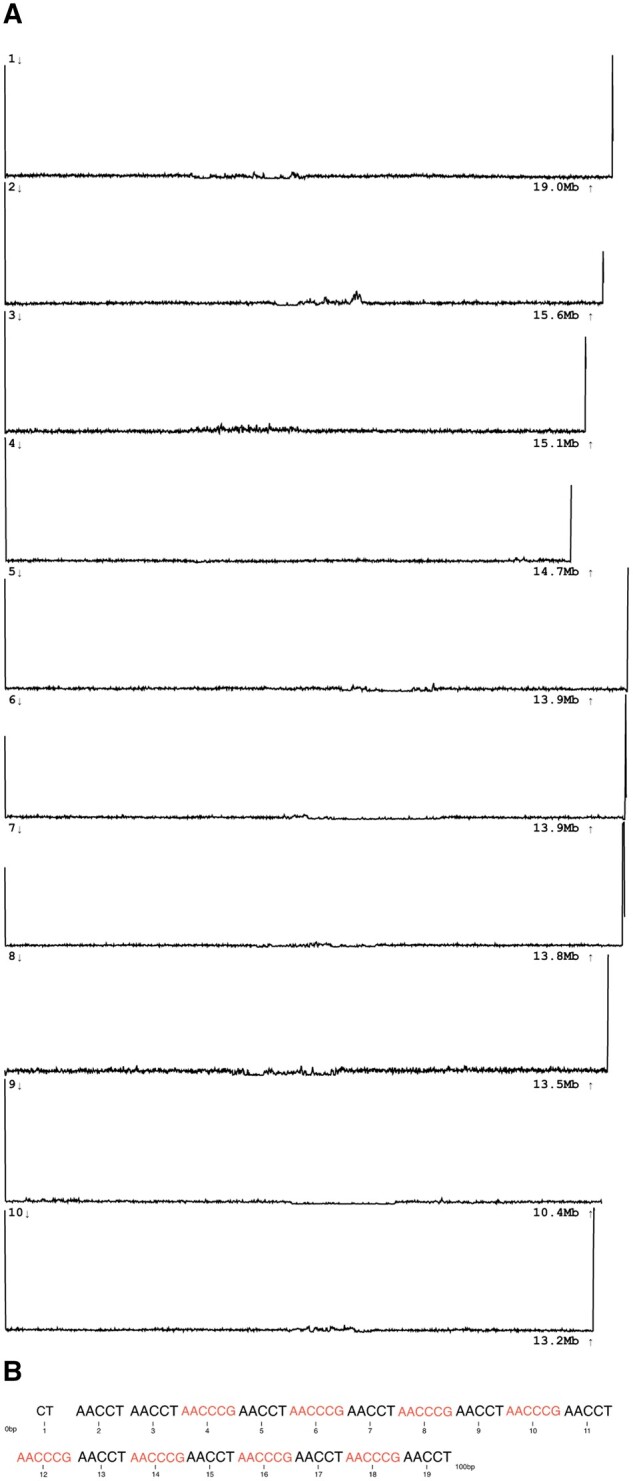
(A) Occurrence of the repeat “AACCT,” which is a substring of the canonical telomeric repeat “AACCCGAACCT,” across the ten largest pseudomolecules (chromosomes) of the Bombus sylvestris genome. In eight molecules, telomeric repeat is found at both ends. Where there are no peaks at the ends of the chromosomes, the telomere has not assembled. The *x*-axis represents the position along each pseudomolecule, while the *y*-axis denotes the frequency of the identified repeat. Plot is generated from “tidk plot.” (B) Sequence composition at the telomeric region of chromosome 1. The first 100 base pairs of chromosome 1 reveal an alternating pattern between the repeats “AACCT” (larger text) and “AACCCG” (smaller text). The repeats are numbered sequentially from 1 to 19, corresponding to their order along the chromosome.

Within the Hymenoptera, the genus *Bombus* presented an unusual compound canonical telomeric repeat structure. “tidk explore” (version 0.2.41) identified a complex telomeric repeat landscape with lexicographically minimal canonical repeats AACCCG, AACCT, and AACCCT pasted together in various combinations. These combinations potentially form HOR’s across the telomeric region. Selecting AACCT as a representative telomeric repeat for *Bombus sylvestris* (Crowley 2023), the distribution of this repeat was visualized along the chromosomes by quantifying repeat occurrences in windows (Fig. 1A). We also display the first 100 base pairs of chromosome 1 to highlight the alternating repeat landscape (Fig. 1B). Eight of the ten molecules displayed have telomeric repeat peaks at each end and are likely to be complete chromosomes. Two lack telomeric repeats at one end, likely because of local incompleteness of the assembly. In the hymenopteran family Vespidae a longer, divergent telomeric repeat, AACCCAGACCC, was conserved across the four vespid genera analyzed (*Vespa*, *Vespula*, *Dolichovespula*, and *Ancistrocerus*).

## 5 Conclusion

tidk is a tool for rapid identification of telomeric repeats in either raw long reads (PacBio or Nanopore) or assembled genomes. Identifying telomeres is crucial in asserting the telomere-to-telomere status of high contiguity assemblies. In addition, we hope that easier telomeric repeat identification will drive telomeric repeat analysis across the tree of life.

We built tidk to aid characterization of telomeric repeats for genomes being assembled as part of the Tree of Life programme, including the Darwin Tree of Life (DToL) project (Darwin Tree of Life Project Consortium 2022). Since version 0.1.5, tidk has been used to investigate and verify telomeric repeats from plants, fungi, and animals across the tree of life (Leonard *et al.* 2022, Yin *et al.* 2022, Erdos *et al.* 2023, Shi *et al.* 2023, Wang *et al.* 2023, Bush *et al.* 2024).

## Supplementary Material

btaf049_Supplementary_Data

## Data Availability

Source code and documentation is available under MIT license at https://github.com/tolkit/telomeric-identifier. Analysis, including simulation and figure generation is available at https://github.com/Euphrasiologist/tidk_paper.
