# Supplementary material for: tidk: a toolkit to rapidly identify telomeric repeats from genomic datasets
Source: Bioinformatics. 2025 Jan 31;41(2):btaf049. doi: 10.1093/bioinformatics/btaf049 (PMC11814493; doi:10.1093/bioinformatics/btaf049)
Supplement: btaf049_Supplementary_Data [file btaf049_supplementary_data.zip › supplementary/Supplementary2_telomere_lengths.html]

tidk SI


# Supplementary S2

All of the following tests were done on an Apple M1 Macbook Pro with 8 cores and 16GB memory.

## Time complexity of `tidk explore`

To explore the performance of `tidk`, we changed the program parameters over fasta files of different
sizes. In this example, we used a minimum kmer length of 5, and a maximum of 30, only considering 0.1% of the length
of the chromosome in from the chromosome ends. You can see that there is a linear increase in time taken for the
program to run given an input fasta size, and around 20 seconds of computation time per gigabase of input DNA. Our
fasta files were generated from copying a genome several times over to
simulate a much larger genome. You can see the code which generated these plots at
https://github.com/Euphrasiologist/tidk\_paper/blob/main/src/complexity.bash.
Running over a kmer range this large is probably too much for most use cases, as most eukaryotes have much shorter
canonical telomere repeat units (<15).

In this next plot, we lower the kmer range to one which is more widely applicable and will use far less
compute, especially for larger genomes. Using these parameters, the program takes around half of the time to run
as for the previous example, around 9.5 seconds per gigabase.

## Time complexity of the algorithms in `tidk search`

`tidk search` is at its heart a simple pattern matching algorithm, so is much faster in general than
`tidk explore` for larger kmer ranges. Speed is around 10 seconds per gigabase of input DNA.
