# Supplementary material for: tidk: a toolkit to rapidly identify telomeric repeats from genomic datasets
Source: Bioinformatics. 2025 Jan 31;41(2):btaf049. doi: 10.1093/bioinformatics/btaf049 (PMC11814493; doi:10.1093/bioinformatics/btaf049)
Supplement: btaf049_Supplementary_Data [file btaf049_supplementary_data.zip › supplementary/Supplementary3_repeat_table.pdf]

# Supplementary Table S3

We simulated genomes, each with a thousand sequence replicates of varying lengths (600, 12,000, or 30,000 nucleotides) and composed of TTAGGG telomeric repeats (with the lexicographically minimal canonical telomeric repeat being AACCCT) at different error rates (1%, 1.5%, 2%, 5%, and 10%). We aimed to assess the performance of `tidk explore` in identifying telomeric repeats.

The table below shows:

1. The simulated conditions (mixture of error rate and sequence length)
2. The most abundant canonical telomeric repeat
3. The count of the most abundant canonical telomeric repeat
4. The most abundant 6-nucleotide canonical telomeric repeat
5. The count of the most abundant 6-nucleotide canonical telomeric repeat
6. The count of AACCCT canonical telomeric repeat
7. The total number of canonical telomeric repeats identified by `tidk explore`

| **Simulation Conditions** | **Most abundant canonical repeat** | **Most abundant count** | **Most abundant 6 nucleotide canonical repeat** | **Most abundant 6 nucleotide count** | **AACCCT count** | **Total canonical repeats** |
|---|---|---|---|---|---|---|
| len12000_err0.0 | AACCCT | 4000 | AACCCT | 4000 | 4000 | 2 |
| len12000_err0.001 | AACCCT | 1014 | AACCCT | 1014 | 1014 | 2 |
| len12000_err0.01 | AAGGT | 1652 | AACCCT | 416 | 416 | 7 |
| len12000_err0.015 | ACCCT | 1896 | AACCCT | 426 | 426 | 7 |
| len12000_err0.02 | AAACC | 1976 | N | 0 | 0 | 8 |
| len12000_err0.05 | ACCAT | 2039 | N | 0 | 0 | 16 |
| len12000_err0.1 | AAGGG | 1776 | N | 0 | 0 | 33 |
| len30000_err0.0 | AACCCT | 10000 | AACCCT | 10000 | 10000 | 2 |
| len30000_err0.001 | AACCCTAACCCT | 738 | AACCCT | 650 | 650 | 2 |
| len30000_err0.01 | AACTT | 5195 | AACCCT | 430 | 430 | 9 |
| len30000_err0.015 | AACCC | 5160 | AACCCT | 412 | 412 | 7 |
| len30000_err0.02 | AACCC | 9896 | AACCCT | 432 | 432 | 7 |
| len30000_err0.05 | AACTT | 4506 | N | 0 | 0 | 28 |
| len30000_err0.1 | AACCC | 3801 | N | 0 | 0 | 43 |
| len600_err0.0 | AACCCT | 200 | AACCCT | 200 | 200 | 2 |
| len600_err0.001 | AACCCT | 200 | AACCCT | 200 | 200 | 2 |
| len600_err0.01 | AACCCTAACCCT | 98 | AACCCT | 46 | 46 | 2 |
| len600_err0.015 | AACCCT | 84 | AACCCT | 84 | 84 | 4 |
| len600_err0.02 | AACCC | 71 | AACCCT | 62 | 62 | 4 |
| len600_err0.05 | ACCCT | 87 | AACCCT | 48 | 48 | 6 |
| len600_err0.1 | AAGGT | 104 | AACCCT | 52 | 52 | 17 |
