# Supplementary material for: tidk: a toolkit to rapidly identify telomeric repeats from genomic datasets
Source: Bioinformatics. 2025 Jan 31;41(2):btaf049. doi: 10.1093/bioinformatics/btaf049 (PMC11814493; doi:10.1093/bioinformatics/btaf049)
Supplement: btaf049_Supplementary_Data [file btaf049_supplementary_data.zip › supplementary/Supplementary1_pseudocode.pdf]

Supplementary File S1

Pseudocode for a **tidk** algorithm used in `tidk explore`. The algorithm is used to merge k-mers with the same minimal form. It explains the code written in the GitHub repository found **here**.

```
function MERGE_KMERS(hashmap)
    merged_hashmap ← {}
    for kmer, count in hashmap.items do
        ▷ Compute the minimal form for this kmer
        minimal_form ← compute_minimal_form(kmer)
        ▷ Merge counts using the minimal form as the key
        if minimal_form in merged_hashmap then
            merged_hashmap[minimal_form] + = count
        else
            merged_hashmap[minimal_form] = count
    return merged_hashmap
```

```
function COMPUTE-MINIMAL-FORM(kmer)
    ▷ Generate all rotations of the kmer
    rotations ← kmer[i:] + kmer[:i] for i in range(len(kmer))

    ▷ Compute reverse complement of the kmer
    reverse_complement ← reverse_complement(kmer)

    ▷ Generate all rotations of the reverse complement
    reverse_rotations ← reverse_complement[i:] + reverse_complement[:i] for i in
    range(len(reverse_complement))

    ▷ Find the lexicographically minimal rotation (smallest in alphabetical order)
    minimal_form ← min(rotations + reverse_rotations)

    return minimal_form
```
